# Disclosing the potential of* Cupressus leylandii* A.B. Jacks & Dallim,* Eucalyptus globulus* Labill.,* Aloysia citrodora* Paláu, and* Melissa officinalis* L. hydrosols as eco-friendly antimicrobial agents

**DOI:** 10.1007/s13659-023-00417-9

**Published:** 2024-01-02

**Authors:** Heloísa H. S. Almeida, Pedro J. L. Crugeira, Joana S. Amaral, Alírio E. Rodrigues, Maria-Filomena Barreiro

**Affiliations:** 1 Centro de Investigação de Montanha (CIMO), Instituto Politécnico de Bragança, Campus de Santa Apolónia, 5300-252 Bragança, Portugal; 2 Laboratório Associado Para a Sustentabilidade Em Regiões de Montanha (SusTEC), Instituto Politécnico de Bragança, Campus de Santa Apolónia, 5300-252 Bragança, Portugal; 3 Laboratory of Separation and Reaction Engineering-Laboratory of Catalysis and Materials (LSRE-LCM), Faculty of Engineering, University of Porto, Rua Dr. Roberto Frias, 4200-465 Porto, Portugal; 4 Associate Laboratory in Chemical Engineering (ALiCE), Faculty of Engineering, University of Porto, Rua Dr. Roberto Frias, 4200-465 Porto, Portugal

**Keywords:** Hydrosols, Essential oil by-products, Chemical composition, Antimicrobial activity, Natural preservatives, Waste valorisation

## Abstract

Antimicrobial resistance is a major global health concern, threatening the effective prevention and treatment of infections caused by microorganisms. These factors boosted the study of safe and green alternatives, with hydrosols, the by-products of essential oils extraction, emerging as promising natural antimicrobial agents. In this context, four hydrosols obtained from *Cupressus leylandii* A.B. Jacks & Dallim, *Eucalyptus globulus* Labill., *Aloysia citrodora* Paláu and *Melissa officinalis* L. were studied. Their chemical composition comprises neral, geranial, 1,8-cineole, terpinen-4-ol, and oplopanonyl acetate, compounds with recognised antimicrobial activity. Concerning antimicrobial activity, significant differences were found using different hydrosol concentrations (10–20% *v/v*) in comparison to a control (without hydrosol), showing the potential of the tested hydrosols to inhibit the microbial growth of *Escherichia coli*, *Staphylococcus aureus*, and *Candida albicans*. *A. citrodora* hydrosol was the most effective one, inhibiting 90% of *E. coli* growth and 80% of *C. albicans* growth, for both hydrosol concentrations (p < 0.0001). With hydrosol concentration increase, it was possible to observe an improved antimicrobial activity with significant reductions (p < 0.0001). The findings of this work indicate the viability of reusing and valuing the hydrosols, encouraging the development of green applications for different fields (e.g., food, agriculture, pharmaceuticals, and cosmetics).

**Graphical Abstract:**

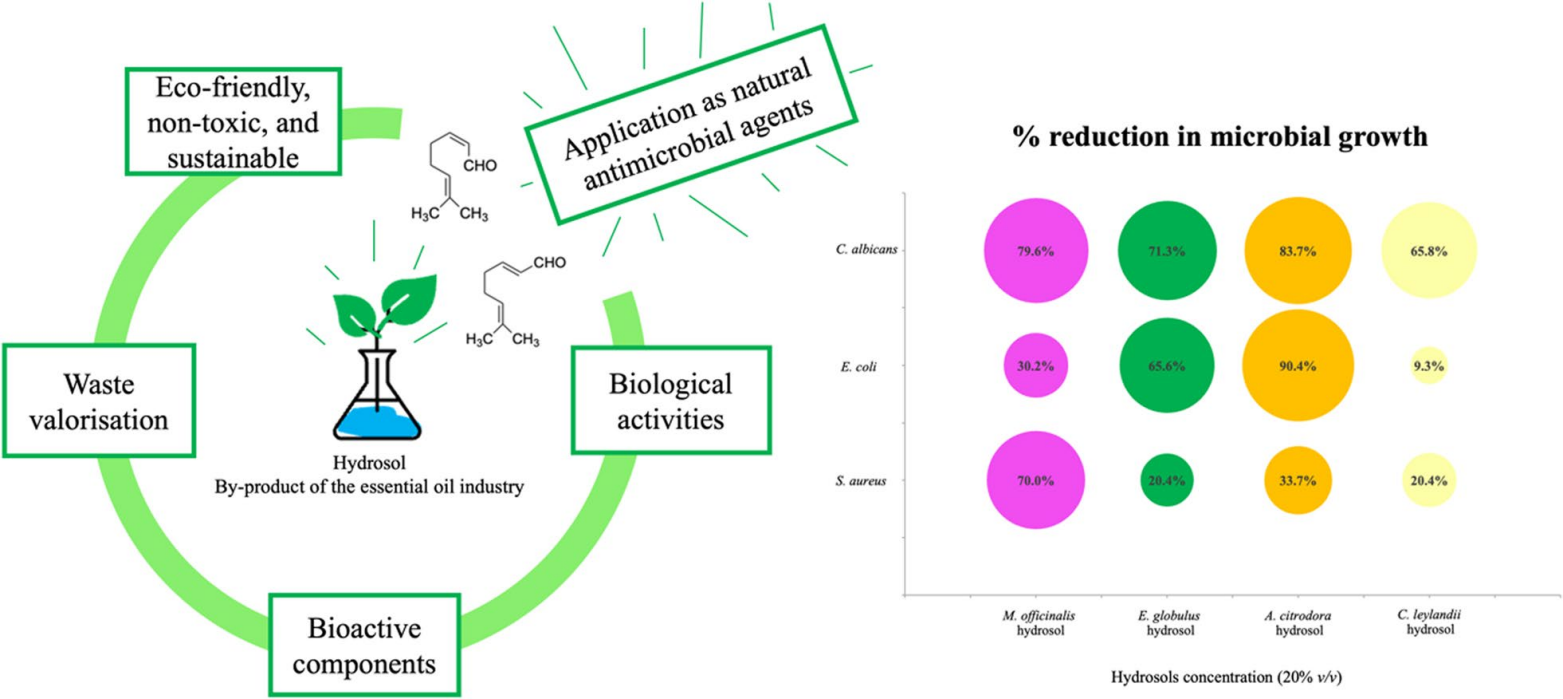

## Introduction

Antimicrobial resistance is a serious threat to public health worldwide, making it harder to effectively prevent and treat chronic illnesses due to the microorganism’s capacity to survive and stay viable in the presence of antibiotics. In addition to the increased morbidity and mortality, this phenomenon may impact different areas, including global health, food sustainability and security, environmental wellness, and socio-economic development [1, 2]. In this scenario, alternative greener, non-toxic, and natural antimicrobial agents with bioactive capacity are needed.

Natural products, especially those derived from plants, have long been used in traditional medicine due to their preservative and therapeutic properties. Their complex chemical composition, which includes alkaloids, flavonoids, phenols, glycosides, steroids, saponins, and terpenoids, wides the sources of molecules with potential antimicrobial capacity. These molecules play a key role in inhibiting the microorganisms, presenting distinctive mechanisms of action that can cause modifications at their metabolic and physiological levels [1, 3, 4]. Several plants have been described as having bioactive properties, including *Melissa officinalis*, a widely used edible medicinal herb from the Lamiaceae family. *M. officinalis* has a phytochemical composition rich in bioactive compounds with pharmacological effects, including antioxidant, antimicrobial, and cytotoxic activities [5]. *Aloysia citrodora*, a species of the Verbenaceae family, often used for medical, cosmetic, and aromatic purposes, has remarkable biological properties like antioxidant, antimicrobial, and antiproliferative activities [6]. In another study [7], the plant genus *Eucalyptus*, which belongs to the Myrtaceae family, is referred to as a valuable source of bioactive components with antioxidant and antimicrobial capacity, making it a useful natural preservative for the pharmaceutical, cosmetical and food applications. The *Cupressaceae*, also known as the cypress family, is a genus of conifers that are found all over the world. Despite having a few species scarcely studied, they are reported to contain important volatile and phenolic compounds in their essential oils (EOs), extracts, and derived compounds. Insecticidal, antibacterial, and antifungal capacities dominate the reported biological activities [8]. These plants, among many others, are known worldwide and used at the industrial level to produce EOs.

Pathogens, including *Staphylococcus aureus* (Gram-positive spherical bacteria), *Escherichia coli* (Gram-negative bacillary bacteria), and *Candida albicans* (yeast), can proliferate in many different niches, allowing them to multiply and spread easily. These commensal microbes potentially cause a wide range of illnesses. For example, *E. coli* may lead to gastrointestinal and extra-intestinal infections, while *S. aureus* and *C. albicans* may affect the skin and mucosae of their hosts, causing systemic infections. In some cases, gastrointestinal intoxications and infections are caused by the development of enterotoxins (*S. aureus*), and Shiga-toxin (*E. coli*), which are ingested through contaminated water, food, and beverages [9–13].

In this scenario, hydrosols, also known as hydrolats, the secondary products of aromatic plant distillation, have raised attention as natural antimicrobials due to their eco-friendly characteristics and bioactive properties. They are a heterogeneous mixture of polar, oxygenated, hydrophilic, and volatile oil components forming hydrogen bonds with water. They contain bioactive hydrophilic substances and few hydrophobic components from the respective EOs, exhibiting bioactivities associated with their chemical composition, namely components holding different functional groups, e.g., methyl, hydroxyl, carbonyl, and carboxyl groups [14–16]. Despite coming from the same process, the composition and efficacy of the two distillation products (oil and hydrosol) vary. EOs application needs caution since several terpene molecules are particularly toxic, irritating skin upon contact. Moreover, they present a strong aroma, which might induce an unpleasant sensation and headaches. For these reasons, they are not typically consumed or used topically. Contrarily, hydrosols, which correspond to dilute terpenic solutions, are less harmful and thus more attractive for these applications [14, 17].

Hydrosols have recently started to receive a lot of credit in a variety of areas, such as food (e.g., flavouring, preservatives, and sanitisers) [18, 19], cosmetic and perfumery [20], aromatherapy [21], agriculture (e.g., biopesticides and repellents) [22], pharmaceutical (e.g., natural antibiotics, antioxidants and anti-inflammatory agents) [17, 23], and medical (e.g., antimicrobial and antitumor agents) [24]. According to circular economy principles, using industrial by-products can be a sustainable way to address the environmental problems caused by waste discharging. This is particularly interesting when they have promising biological properties such as antioxidant, anti-inflammatory, and antimicrobial activities [25–27].

To reduce the lack of knowledge regarding hydrosols, the present work aimed to study the chemical composition of four hydrosols (*Cupressus leylandii* A.B. Jacks & Dallim, *Eucalyptus globulus* Labill., *Aloysia citrodora* Paláu and *Melissa officinalis* L.), obtained by hydro-distillation, and determine the antimicrobial properties against three pathogenic microorganisms, *Staphylococcus aureus*, *Escherichia coli*, and *Candida albicans*. It is expected to contribute to the recovery and use of these by-products finding eco-friendly applications as natural antimicrobial agents.

## Results and discussion

### Visual, olfactory, and acidity attributes of hydrosols

Hydrosols are mentioned in the literature as diluted solutions with an acidic character, presenting different characteristics such as aroma, colour, and chemical composition [16, 28]. In this work, all hydrosols were identified as a colourless liquid with mild to strong aroma and acidic pH as described in Table 1.

**Table 1:** pH and sensorial properties of hydrosols

Hydrosol	Main characteristics	pH value
*C. leylandii*	Colourless liquid with a mid-scent	2.9
*E. globulus*	Colourless liquid with a strong scent	3.2
*A. citrodora*	Colourless liquid with a strong scent	4.1
*M. officinalis*	Colourless liquid with a mid-scent	3.2

The pH values for the studied hydrosols ranged from 2.9 to 4.1, which agrees with other published works [16, 29] reporting pHs between 2.2 and 5.5, thus corroborating the predominantly acidic nature of these products. Jakubczyk and co-workers [30], who investigated the 17 most popular hydrosols for the cosmetic market, found a pH value of 3.34 for *Melissa officinalis* hydrosol, similar to the value obtained in this work (3.2). pH is an important parameter affecting hydrosols’ final application, including its therapeutic effects [31].

### Chemical composition of hydrosols

Table 2 provides the complete chemical composition of the studied hydrosols, where the identified components are mainly oxygenated monoterpenes. Following EOs extraction, the oil phase enters in contact with the water phase, allowing different polar hydrophilic volatile compounds to form hydrogen bonds and disperse in the water phase (hydrosol). The degree of hydrogen bonding of the components with water molecules is determined by the component’s chemical structure (polarity factor), explaining why the oxygenated compounds present relatively higher solubility in water (when compared to hydrocarbons, for example), thus appearing as major components in the studied hydrosols [28, 32]. Besides that, a range of factors, including environmental (e.g., temperature, rainfall), geographical origin, harvesting conditions (e.g., season, growth stage), and plant material post-harvesting processing (e.g., drying, extraction methods, distillation conditions), influences the composition of the essential oil and respective by-products (content and quality) [28, 33].

**Table 2:** Chemical composition of volatile compounds present in the hydrosols extracted from *C. leylandii*, *E. globulus*, *A. citrodora*, and *M. officinalis*, by hydro-distillation (mean ± SD, n = 3)

Compound	RT	LRI^a^	LRI^b^	*C. leylandii*	*E. globulus*	*A. citrodora*	*M. officinalis*
				Relative %	Relative %	Relative %	Relative %
2*E*-Hexenal	10.10	847	846	–	0.42 ± 0.19	–	1.27 ± 0.16
Isopentyl acetate	11.29	872	869	–	0.022 ± 0.005	–	–
4-Mercapto-4-methyl-pentan-2-one	14.46	936	–	–	0.024 ± 0.01	–	–
1-Octen-3-ol	16.52	976	974	0.60 ± 0.02	–	0.80 ± 0.02	0.42 ± 0.09
3-Methyl-3-cyclohexen-1-one	16.73	979	940	–	0.11 ± 0.02	–	–
Sulcatone	16.91	983	981	–	–	0.66 ± 0.15	0.27 ± 0.02
3-Octanol	17.34	991	988	–	–	0.37 ± 0.13	–
*p*-Cymene	18.63	1016	1020	–	–	–	0.054 ± 0.007
Limonene	18.94	1023	1024	0.23 ± 0.03	–	0.14 ± 0.01	0.14 ± 0.01
1,8-Cineole	19.15	1026	1026	0.9061 ± 0.0003	**90.12 ± 1.01**	4.58 ± 0.62	2.21 ± 0.02
Benzeneacetaldehyde	19.77	1038	1036	–	–	–	0.45 ± 0.01
*cis*- Linalool oxide	21.23	1066	1067	–	0.12 ± 0.02	0.14 ± 0.03	–
Fenchone	21.98	1081	1083	3.32 ± 0.04	–	–	–
*trans*- Linalool oxide	22.05	1082	1084	–	0.066 ± 0.006	–	–
Linalool	22.66	1094	1095	0.122 ± 0.007	0.039 ± 0.006	0.51 ± 0.11	0.27 ± 0.07
Fenchol	23.31	1107	1107	0.367 ± 0.006	–	–	–
*trans*-*p*-Mentha-2,8-dien-1-ol	23.67	1114	1119	2.95 ± 0.22	–	0.09 ± 0.02	–
*α*-Campholenal	23.94	1119	1122	–	0.069 ± 0.009	–	–
*cis*-*p*-Mentha-2,8-dien-1-ol	24.40	1128	1133	1.551 ± 0.014	0.026 ± 0.002	–	–
*trans*- Pinocarveol	24.58	1131	1135	-	0.168 ± 0.002	–	–
*trans*-*p*-Menth-2-en-1-ol	24.60	1133	1136	0.76 ± 0.06	–	–	–
Camphor	24.81	1137	1141	8.55 ± 0.27	–	–	–
*cis*- Verbenol	24.89	1137	1137	–	0.03 ± 0.01	–	–
*trans*- Verbenol	24.89	1137	1140	–	0.26 ± 0.03	–	–
*exo*- Isocitral	24.92	1139	1140	–	–	0.80 ± 0.08	0.18 ± 0.02
Camphene hydrate	25.03	1141	1145	1.05 ± 0.31	–	–	–
*β*-Pinene oxide	25.52	1151	1154	0.37 ± 0.04	–	–	–
Pinocarvone	25.75	1156	1160	0.43 ± 0.09	0.0271 ± 0.0002	–	–
Isoneral	25.88	1159	1160	–	–	0.96 ± 0.27	0.36 ± 0.06
Borneol	25.92	1159	1165	0.98 ± 0.03	–	–	–
*δ*-Terpineol	25.99	1160	1162	–	0.42 ± 0.01	0.31 ± 0.14	–
Umbellulone	26.28	1167	1167	0.33 ± 0.02	–	–	–
Terpinen-4-ol	26.50	1171	1174	**36.20 ± 0.14**	1.04 ± 0.09	0.49 ± 0.11	–
Isogeranial	26.80	1177	1177	–	–	–	1.44 ± 0.02
*p*-Cymen-8-ol	26.90	1179	1179	2.33 ± 0.53	0.18 ± 0.01	–	–
*α*-Terpineol	27.15	1184	1186	6.869 ± 1.004	6.03 ± 0.60	1.64 ± 0.05	–
Myrtenol	27.44	1190	1194	0.98 ± 0.24	0.077 ± 0.006	–	–
*trans*-Isopiperitenol	27.66	1194	1192	–	–	0.23 ± 0.07	–
*trans*-Dihydro carvone	27.80	1197	1200	0.41 ± 0.07	–	–	–
Verbenone	28.06	1203	1204	–	0.045 ± 0.003	–	–
*trans*-Carveol	28.54	1213	1215	–	0.07 ± 0.01	–	–
Oxiranecarboxaldehyde, 3-methyl-3-(4-methyl-3-pentenyl)-	28.71	1216	1215	–	–	0.36 ± 0.02	–
Citronellol	28.91	1221	1223	–	–	–	0.09 ± 0.02
Nerol	28.95	1222	1227	–	–	2.85 ± 0.12	-
*β*-citronellol	28.98	1222	1223	–	–	–	0.39 ± 0.09
Neral	29.54	1234	1235	–	0.064 ± 0.009	**39.01 ± 0.94**	**42.027 ± 0.003**
Geraniol	30.20	1248	1249	–	–	1.03 ± 0.16	–
Geranial	30.93	1264	1264	–	–	**38.91 ± 2.15**	**50.08 ± 0.02**
Methyl myrtenate	32.18	1291	1292	4.13 ± 0.47	–	–	–
exo-2-Hydroxycineole acetate	34.20	1336	1342	–	0.24 ± 0.02	–	–
*α*-Terpinyl acetate	34.54	1343	1344	0.114 ± 0.003	–	–	–
*E*-Caryophyllene	37.53	1412	1417	–	–	–	0.13 ± 0.03
γ-Muurolene	40.12	1474	1478	–	–	–	0.07 ± 0.02
Spathulenol	44.11	1574	1577	–	–	0.67 ± 0.05	–
(−)-Globulol	44.39	1581	1590	–	0.050 ± 0.001	–	–
*β*-Oplopenone	45.32	1605	1606	0.424 ± 0.044	–	–	–
Oplopanone	49.92	1742	1739	0.526 ± 0.024	–	–	–
Oplopanonyl acetate	52.61	1882	1885	**12.76 ± 0.78**	–	–	–
Total identified				88.11 ± 0.04	99.72 ± 0.01	96.20 ± 0.41	99.442 ± 0.004
Monoterpene hydrocarbons				13.09 ± 0.04	0.213 ± 0.001	0.14 ± 0.01	0.193 ± 0.008
Oxygen-containing monoterpenes				60.65 ± 0.83	98.88 ± 0.21	93.55 ± 0.92	97.09 ± 0.11
Sesquiterpenes hydrocarbons				–	0.050 ± 0.001	0.67 ± 0.05	0.21 ± 0.05
Oxygen-containing sesquiterpenes				13.71 ± 0.84	–	–	–
Others				0.60 ± 0.02	0.57 ± 0.19	1.83 ± 0.30	1.96 ± 0.05

RT = retention time; LRI^a^ = linear retention index determined on a SH-RXi-5 ms fused silica column relative to a series of n-alkanes (C8–C40); LRI^b^ = linear retention index reported in the literature [42]; Relative % is given as mean ± SD, n = 3

**Fig. 1:**
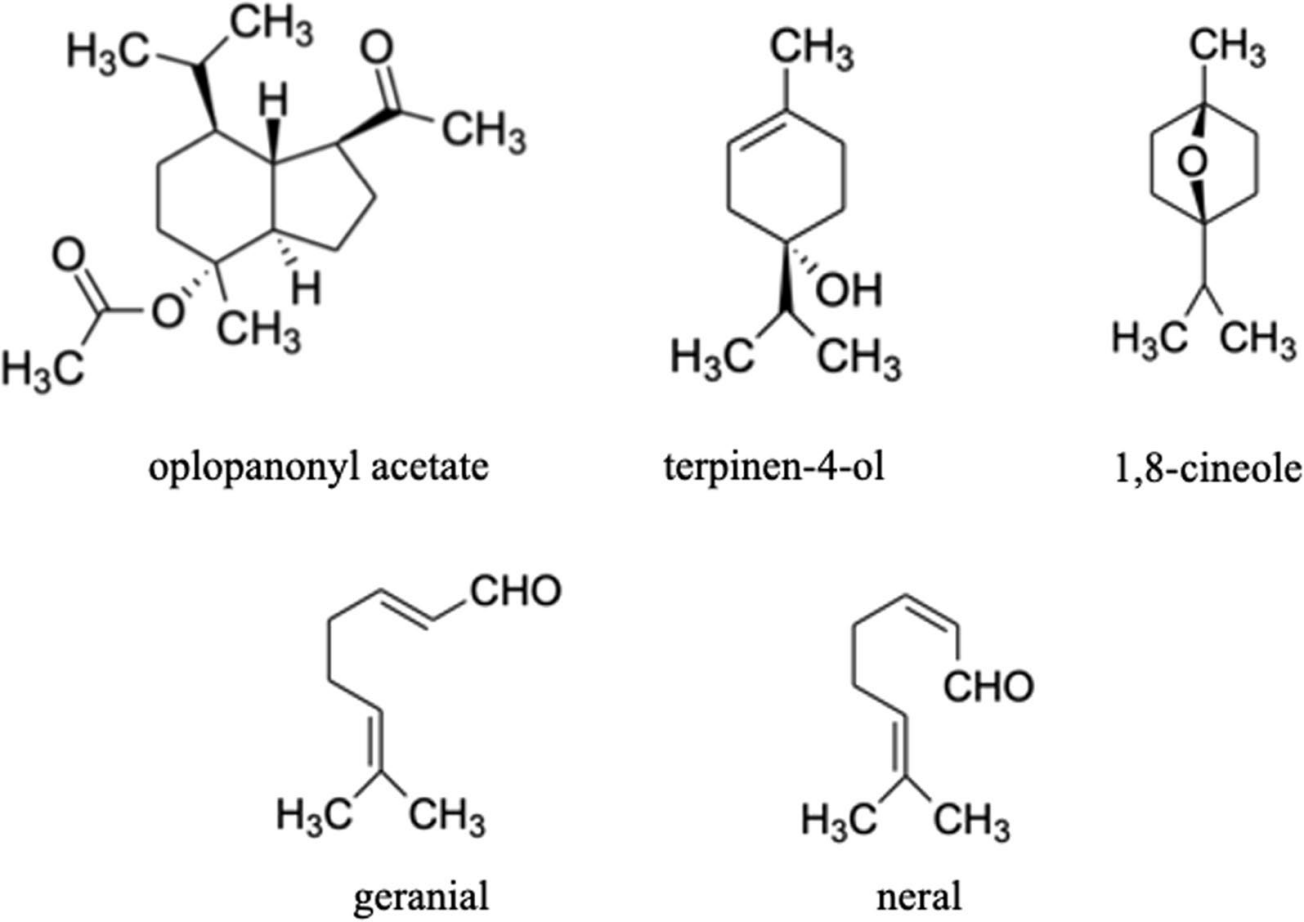
Chemical structures of the major compounds identified in the studied hydrosols (*C. leylandii*, *E. globulus*, *A. citrodora*, and *M. officinalis*)

In *C. leylandii* hydrosol, 88.1% of the compounds were identified, with terpinen-4-ol (36.2%) and oplopanonyl acetate (12.8%) as the two major components. Concerning the hydrosol of *E. globulus,* 99.7% of the constituents were identified, with 1,8-cineole (90.1%) as the predominant one. The main components of the *A. citrodora* and *M. officinalis* hydrosols (with 96.6% and 99.4% of the total compounds identified, respectively) were citral isomers known as geranial (38.9% and 50.1% for *A. citrodora* and *M. officinalis,* respectively) and neral (39.0% and 42.0% for *A. citrodora* and *M. officinalis,* respectively). Low concentrations of less than 10% were determined for all the other components identified in the studied hydrosols. Figure 1 presents the chemical structure of the major compounds present in the hydrosols (e.g., oxygen-containing sesquiterpenes (oplopanonyl acetate) and oxygen-containing monoterpenes (terpinen-4-ol, 1,8-cineole, geranial, and neral)).

Although the composition of *C. leylandii* and *A. citrodora* essential oils can be found in the literature, no previous works evaluated their hydrosol’s chemical composition, with this data being reported for the first time in this work; even though *C. leylandii* hydrosol presents the same main component (terpinen-4-ol) as the hydrosols of the cypress family, such as the ones obtained from the *C. lusitanica* and *C. sempervirens* species [34, 35]. Moreover, the main components of *A. citrodora* hydrosols (neral and geranial) are likewise found in the corresponding essential oils [36, 37]. In other studies [38–40], the volatile composition of hydrosols from *Eucalyptus* species was studied, being found that oxygenated monoterpenes, particularly 1,8-cineole, are present in most of these hydrosols. This fact was also verified in this work, where this compound represents 90.1% of the *E. globulus* hydrosol composition. Petrakis and collaborators [41], who analysed the composition of *M. officinalis* hydrosols, reported that their major compounds were carvacrol, neral and geranial, analogously to this work, except for carvacrol, whose absence may be related to environmental factors and geographical origin of the studied plant species.

Studying a hydrosol’s chemotype is fundamental to understanding the biological mechanisms underlying its bioactivity and directing its use to a particular application [14]. Although, currently, there are no quality standards for this class of natural products in the global pharmacopoeias, their standardisation (chemical and biological characterisation) will help ensure their quality, prospective uses, and safety [25, 27].

### Antimicrobial activity of hydrosols

**Fig. 2:**
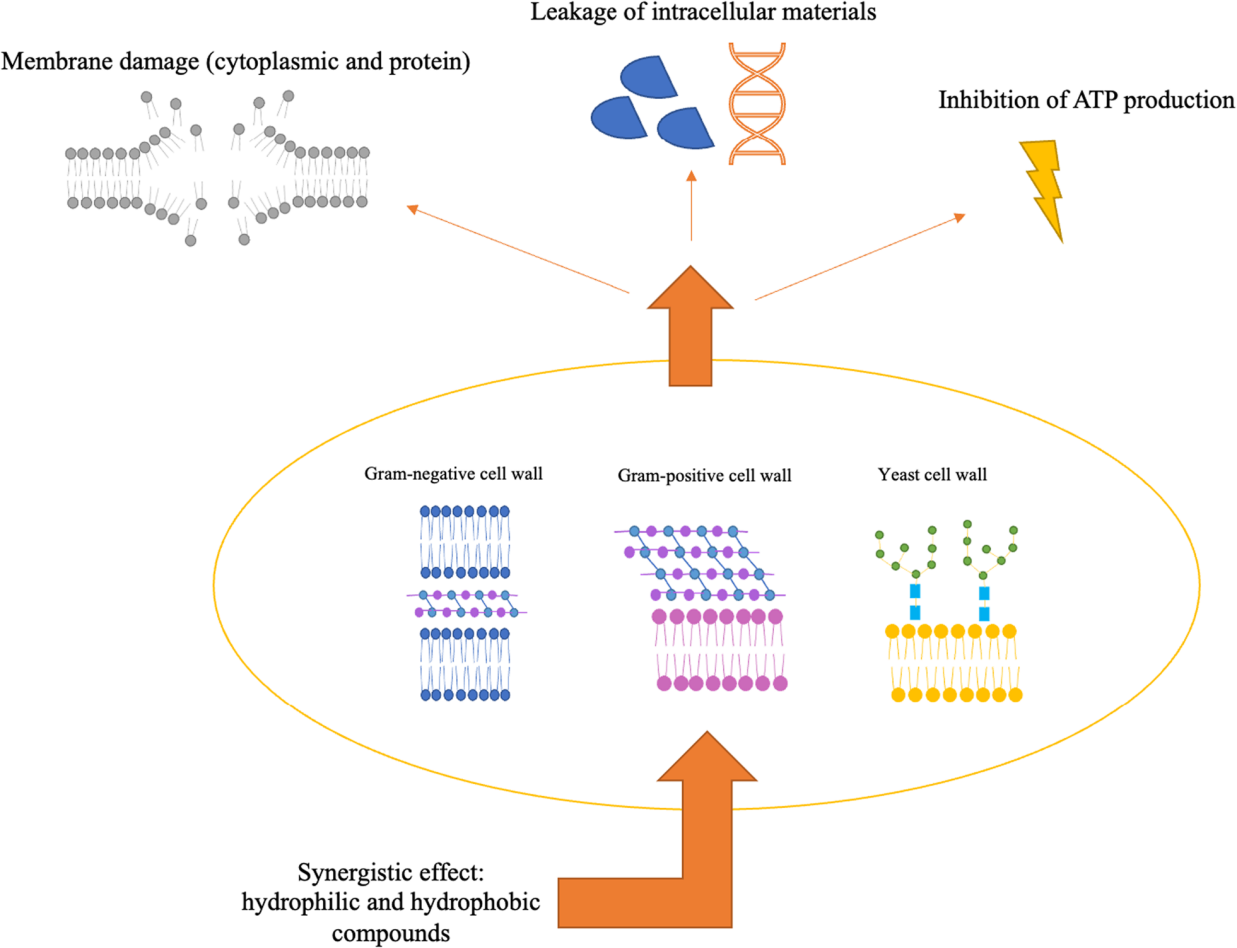
Mechanisms of action of hydrosol components on different cell walls (Gram-negative, Gram-positive, and yeast cells) bosting antimicrobial activity

The chemical composition of a hydrosol indicates whether it has the potential to suppress microbial growth or pro-oxidant and inflammatory processes. Even though the potential mechanisms behind plant antimicrobial effects are not fully understood, these processes are attributed to the synergistic interaction established by compounds in their composition, which might have unique functional groups, polarities, and bioactivities. The interaction between these components and the bacterial cell membrane defines how antimicrobial activity occurs through different action mechanisms (Fig. 2) [43–45]. Depending on whether the bacteria is Gram-positive or Gram-negative, different areas of the microbial cells might be involved. Their susceptibility differs since Gram-positive bacteria contain a thick peptidoglycan layer connected to other hydrophobic compounds. This hydrophobic layer surrounding Gram-positive bacteria may facilitate the entrance of hydrophobic compounds. Gram-negative bacteria, conversely, have a more intricate cell wall, consisting of an outer membrane linked by lipoproteins to the inner peptidoglycan layer, increasing the resistance to the crossing of hydrophobic compounds [46, 47].

**Fig. 3:**
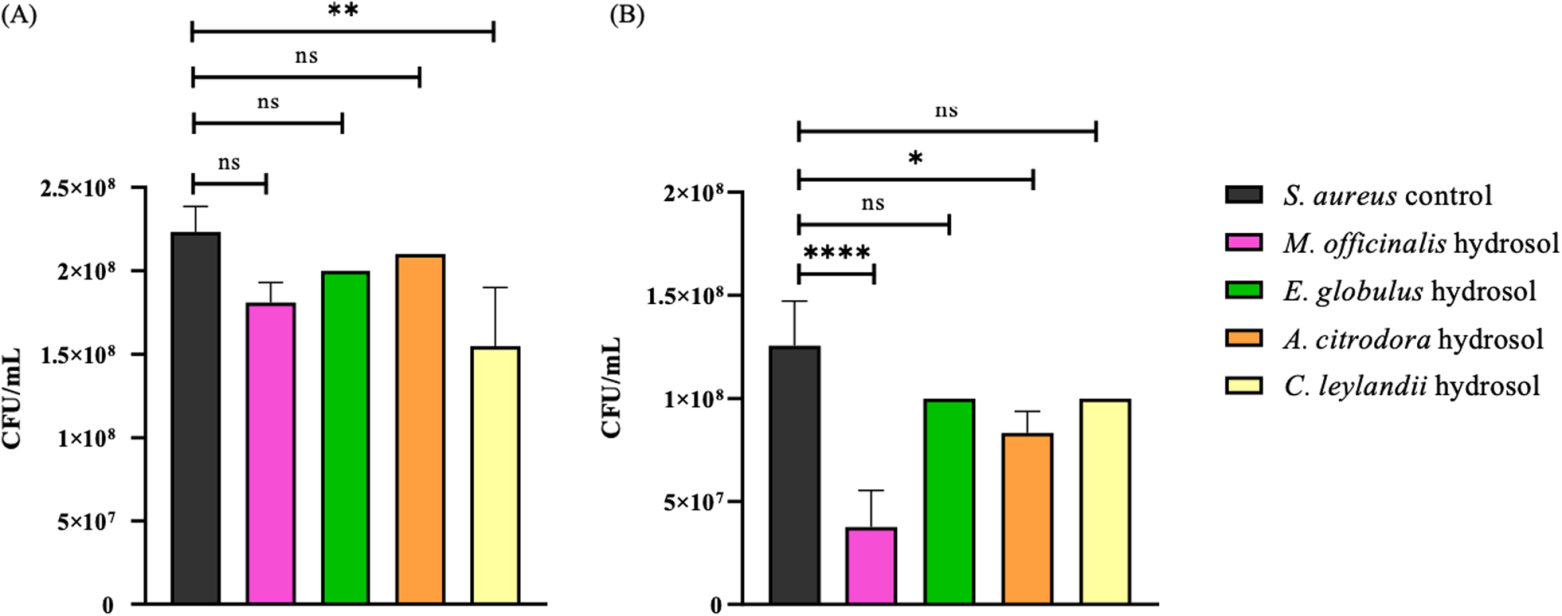
Quantification of *S. aureus* (CFU/mL) in the studied hydrosols, where (**A**) 10% hydrosol concentration and (**B**) 20% hydrosol concentration. ****p < 0.0001; ***p < 0.001; **p < 0.01; *p < 0.05; ns means not significant

**Fig. 4:**
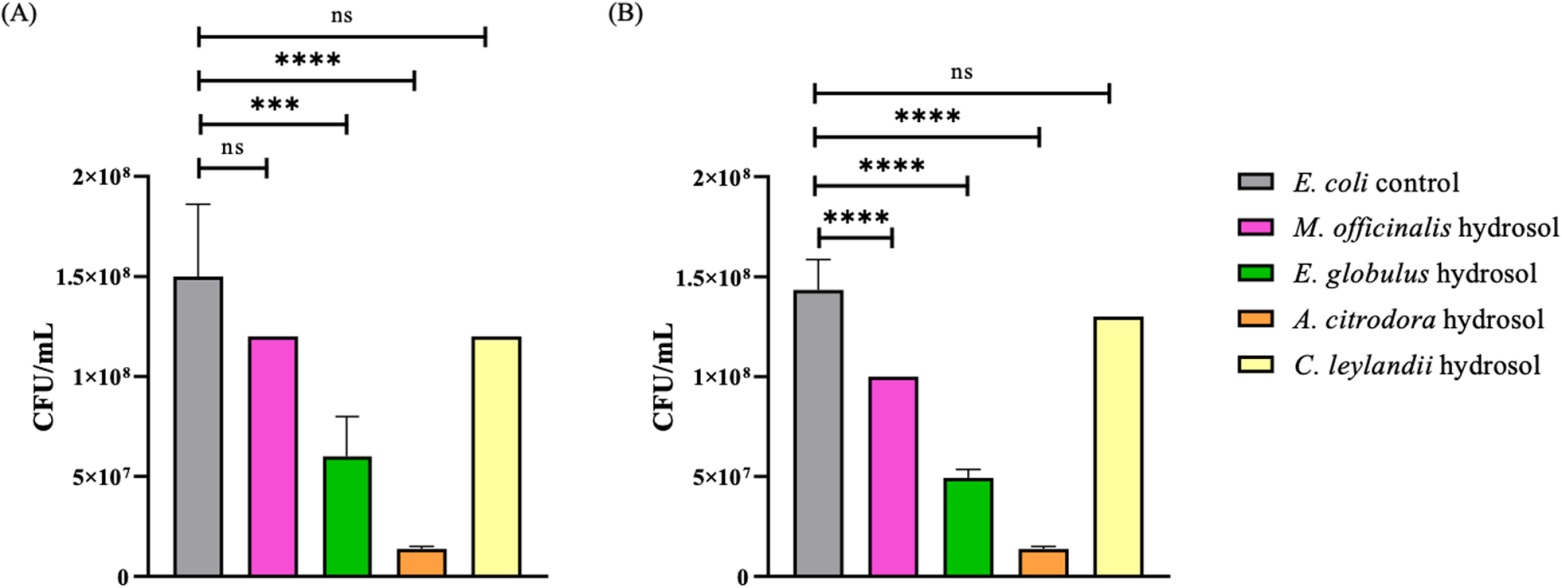
Quantification of *E. coli* (CFU/mL) in the different studied hydrosols, where (**A**) 10% of hydrosol concentration and (**B**) 20% hydrosol concentration. ****p < 0.0001; ***p < 0.001; **p < 0.01; *p < 0.05; ns means not significant

**Fig. 5:**
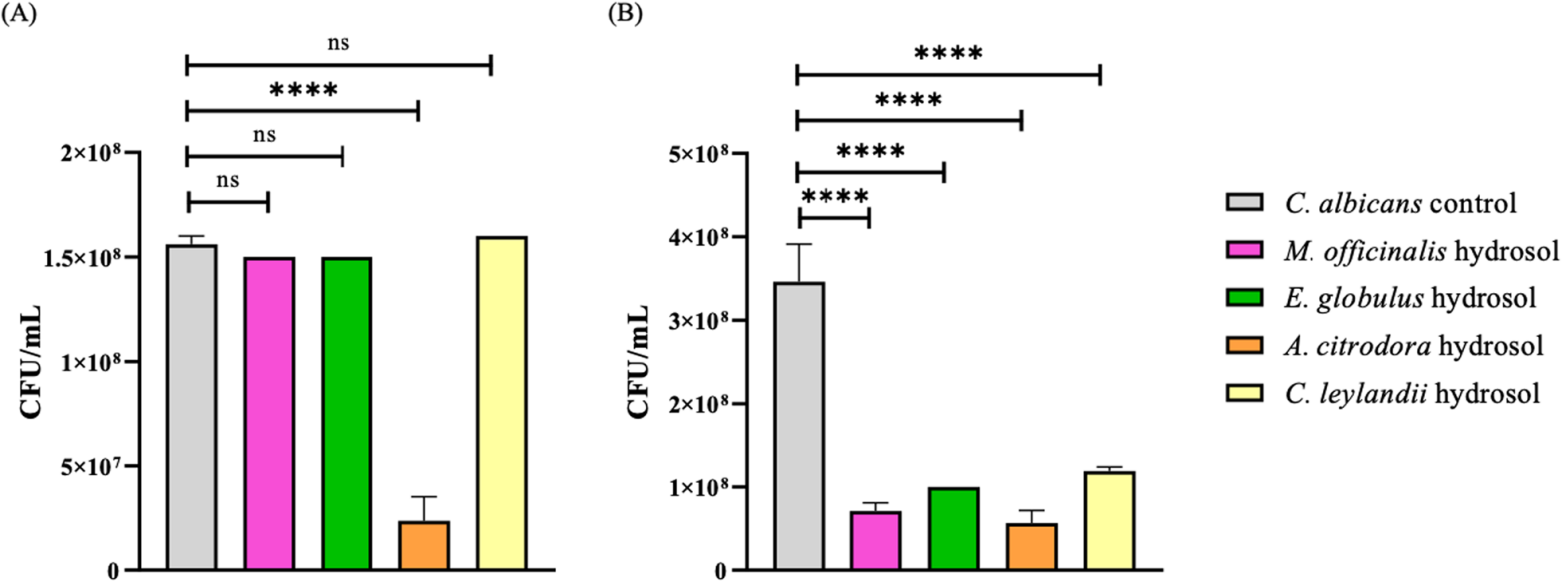
Quantification of *C. albicans* (CFU/mL) in the different studied hydrosols, where (**A**) 10% of hydrosol concentration and (**B**) 20% hydrosol concentration. ****p < 0.0001; ***p < 0.001; **p < 0.01; *p < 0.05; ns means not significant

According to the literature, the major components identified in the studied hydrosols (neral, geranial, 1,8-cineole, terpinen-4-ol, and oplopanonyl acetate) are associated with antimicrobial activity [48–50], corroborating the results of the performed antimicrobial assays. In this work, the statistical analysis enabled determining when there was a significant difference between the samples containing the hydrosol and a control (a sample prepared without adding hydrosol), proving whether the hydrosols can inhibit microbial growth. These differences are evident in Figs. 3, 4 and 5, where the effect of the studied hydrosols on microbial growth is represented. Analysing the susceptibility of *S. aureus* to the studied hydrosols (Fig. 3), it was verified that the concentration of 10% led to a reduction of 29.9% (p < 0.01) when using *C. leylandii* hydrosol, with no significant reduction when the other hydrosols were used. For 20%, the effect against *S. aureus* was increased, namely by lowering the microbial growth by 70.0% (with a significance of p < 0.0001) and 33.7% (p < 0.05) for the *M. officinalis* and *A. citrodora* hydrosols, respectively, compared to the control.

The *A. citrodora* hydrosol was the most effective against *E. coli*, with reductions of 90.8% (at 10% concentration) and 90.4% (at 20% concentration) with a significant difference of p < 0.0001 for both concentrations, which represents a reduction of 1 log10. The effect of this hydrosol was followed by *E. globulus* with cuts of 60% (at 10% concentration with p < 0.001) and 65.6% (at 20% concentration with p < 0.0001), and *M. officinalis*, which was able to reduce 30.2% when using 20% concentration (p < 0.0001) (Fig. 4).

*A. citrodora* hydrosol was also the most effective in inhibiting yeast growth, with reductions of 84.8% (at 10% concentration) and 83.7% (at 20% concentration) for *C. albicans,* with a significance of p < 0.0001 for both concentrations. The other hydrosols only showed this bioactivity for 20% concentration, with reductions of 79.6% for *M. officinalis*, 71.3% for *E. globulus,* and 65.8% for *C. leylandii* (Fig. 5). A significant difference of p < 0.0001 was observed for all hydrosols compared to the *C. albicans* control.

The findings of this work reveal that the antimicrobial activity of hydrosols often rises with concentration, with *A. citrodora* and *M. officinalis* as the most promising ones, followed by *E. globulus* and *C. leylandii* hydrosols. In this way, analysing the chemical composition of the two most promising hydrosols, their antimicrobial capacity may be associated with the main components’ composition, the isomers of citral, which have been shown to have both biofilm-preventing and antimicrobial properties [51, 52]. According to Viktovorá and collaborators [53], who investigated the microbial cells’ resistance to citral, found MIC (minimal inhibition concentration) values of 110 and 92 μL/L on *C. albicans* and *S. aureus*, respectively, being able to inhibit both bacteria and yeast growth. Chueca et al. [54] reported that a concentration of 300 μL/L of citral could inactivate at least 2.5 log10 cycles of exponentially growing cells of *E. coli*. Results reported by Somolinos et al. [55] showed that citral treatment caused sublethal injury to the cytoplasmic and outer membranes of *E. coli* cells. Citral mode of action may involve the penetration in phospholipid membranes, physical disruption of structural and functional characteristics, interruption of electron transfer across membranes, and oxidative stress culminating in membrane lipid peroxidation (leading to a burst on reactive oxidative species) [52, 56]. Thus, the present data may support the efficacy found in this work for the *A. citrodora* and *M. officinalis* hydrosols against the studied microorganisms. However, it is important to note that this major compound is diluted in a mixture of the hydrosol compounds. The antimicrobial potential may come from a synergistic effect among this by-product’s different compounds and citral.

Regarding hydrosols’ bioactivities, in the study of Hung et al., [26], *L. cubeba* fruit hydrosol containing neral and geranial as major components, inhibited the proliferation of *C. albicans* and revealed a fungicidal activity by applying it at 10 and 40% (*v/v*) concentration, respectively. These findings corroborate the results of this work where *C. albicans* was inhibited by approximately 80% with a significance of p < 0.0001 by applying 10–20% (*v/v*) of *A. citrodora* hydrosol, and 20% (*v/v*) of *M. officinalis* hydrosol, both hydrosols holding the main components reported for the *L. cubeba* hydrosol.

1,8-cineole has also been reported as a strong antimicrobial bioactive [48, 49]. This property can be related to its mode of action, which involves irreversible damage to the cell membrane leading to a decrease in ATP (adenosine triphosphate), protein, and DNA (deoxyribonucleic acid), as well as to cytoplasmic leakage [57]. Moreover, in the study by Khalaf and co-workers [58], *Eucalyptus calmadulensis* hydrosols, which present 1,8-cineole as the main compound, showed to inhibit different bacteria, among them *E. coli*, *P. aeruginosa*, *S. epidermidis*, *S. mutans*, *K. pneumoniae*, *P. vulgaris* (when applied directly, 100% concentration), and *P. syogenes* (with 25% concentration). In the present work, it was possible to reduce 60–65% of *E. coli* growth using lower concentrations (10–20% (*v/v*)) of *E. globulus hydrosol*, indicating a promising result for more dilute applications of this by-product. Even presenting the lowest found bioactivity, some authors have reported that the main compounds of *C. leylandii* hydrosol (terpinen-4-ol and oplopanonyl acetate) also have antimicrobial potential [48, 50]. These compounds can penetrate cell walls and membranes, causing internal osmotic pressure, weakening and rupturing the membrane and, subsequently, losing the cytoplasmic material [59]. Although the precise mechanisms underlying the oxygen-containing terpene groups’ antimicrobial potential remain unclear, their lipophilic nature often results in cellular membrane expansion and damage, causing an increase in permeability, disruption of membrane-bound proteins, respiration suppression, and altered ion transport [60].

Various factors can influence a compound’s biological activity, with functional groups holding different impacts by playing a role in polarity, solubility, and hydrogen bonding capacity, among others. The bioactivity of oxygenated molecules and hydrocarbons follows the following order: phenols > aldehydes > ketones > alcohols > ethers > hydrocarbons [61, 62]. In this regard, hydrosols’ antimicrobial activity might be favoured by their hydrophilic environment, which increases the volatiles’ bioavailability for interaction with bacteria and fungi. Particularising, a compound’s polarity will affect its capacity to permeate and/or disrupt membranes. As a result, cellular targets of more hydrophobic compounds (acting on membrane disruption) differ from those of less hydrophobic molecules (acting on interactions with proteins) [17, 62]. In the study of Buccioni et al. [63], *L. monocytogenes* cells treated with 500 μL/mL of *Coridothymus capitatus* hydrosols, showed a diffuse aggregation and cell damage in response to the implied stress. These factors may indicate a synergistic effect among hydrosol components able to promote cellular stress, even when they show, individually, low antimicrobial activity. These findings point out a promising use of hydrosols in inhibiting target microorganisms in different environments.

## Conclusions

Chemical and antimicrobial characterisation of hydrosols derived from plants used in EOs industries (*C. leylandii*, *E. globulus*, *A. citrodora*, and *M. officinalis*) was performed. Their main components were identified, and the associated antimicrobial potential was disclosed, considering the use of hydrosols as natural preservatives. Among the studied hydrosols, it was possible to recognise the most promising ones as *A. citrodora* > *M. officinalis* > *E. globulus* > *C. leylandii*, based on the antimicrobial capacity evaluation, which showed significant differences compared to the control (sample with no added hydrosol).

*A. citrodora* hydrosol used at 10% and 20% (*v/v*) concentrations was able to inhibit 90% of *E. coli* and 80% of *C. albicans* growth (with p < 0.0001), indicating its potential as an antimicrobial agent. The findings of this work revealed that the antimicrobial activity of hydrosols increased with concentration, presenting significant reductions (p < 0.0001), namely of 70% and 79.6% on *S. aureus* and *C. albicans* growth, respectively, with *M. officinalis* hydrosol, and 71.3% on *C. albicans* with *E. globulus* hydrosol.

Overall, hydrosols from aromatic plants, and even forestry biomass, may add value to productive chains, contributing to the consolidation of a biobased circular economy, by transforming this by-product into a green, non-toxic, and valuable ingredient for numerous applications in areas such as food, agriculture, pharmaceuticals, and cosmetics. Hydrosols are thus emerging as relevant candidates for antimicrobial applications, finding a direct use without prior pre-treatments, which also conforms with the principles of green chemistry.

## Experimental section

### General experimental procedures

Four different plants (*Cupressus leylandii* A.B. Jacks & Dallim, *Eucalyptus globulus* Labill., *Aloysia citrodora* Paláu and *Melissa officinalis* L.) supplied by Deifil Technology Lda (Póvoa de Lanhoso, Portugal), were received fresh, cut (leaves, branches, and flowers), frozen, and stored at − 20 °C (Hotpoint-Ariston, Italy). HPLC grade n-hexane (CarloErba Reagents, France) and anhydrous sodium sulphate (Sigma-Aldrich, Germany) were used in sample preparation for the chemical analysis. The microbial cultures (bacteria, yeast) selected in this study (*Staphylococcus aureus* ATCC 6538, *Escherichia coli* ATCC 8739, and *Candida albicans* ATCC 10231) were purchased from Mistracon (Spain). The microorganisms’ substrates, brain–heart infusion (BHI) broth and nutrient agar were purchased from Liofilchem (Italy). The used water was distilled water.

### Hydrosols obtainment

The hydrosols were obtained by hydro-distillation using a plant mass-to-water ratio of 1:1 (*w/w*), following an adapted procedure [64]. Briefly, 50 g of the plant were weighed and charged to the distillation vessel, then added with 50 mL of distilled water. The procedure comprised a first heating period to reach the water’s boiling point. From this point, the hydrosols were collected for 10 min. After cooling, the final hydrosols were stored under refrigerated conditions at 4 °C before analysis. Right after production, the hydrosols were examined. The visual and olfactory inspection, done by one individual, was performed to preliminary access colour and odour sensory parameters. The pH was evaluated using a pH meter (InoLab, WTW Series pH 720, Weilheim, Germany).

### Hydrosols chemical composition

The chemical composition of hydrosols was characterised by gas chromatography-mass spectrometry (GC–MS, Shimadzu, Japan) analysis. The sample preparation comprised a liquid–liquid extraction (LLE), where the hydrosol samples (15 mL) were vigorously mixed with 5 mL of n-hexane in a separating funnel for approximately 10 min. After phase separation, the lower-density liquid (n-hexane phase) was collected, added with anhydrous sodium to remove water, and filtered (Whatman filter n°4). The used gas chromatography conditions followed the ones previously described in [65] using an SH-RXi-5 ms column system (30 m × 0.25 mm × 0.25 μm). The injector temperature was set at 260 °C. The oven temperature programming was as follows: 40 °C for 4 min, raised to 175 °C at a rate of 3 °C/min, then to 300 °C at a rate of 15 °C/min and held for 10 min. The sampling method used a split ratio of 1:10, and the injection volume was 1 μL. Helium was applied as the carrier gas adjusted to a linear velocity of 30 cm/s. The ionisation energy was 70 eV, and a scan range of 35–500 u with a scan time of 0.3 s was used. The compounds were identified by comparing the linear retention index (LRI) and the mass spectra with the NIST17 mass spectral Library data (considering a similarity > 90%). LRI determination was based on the retention times obtained from a mixture of *n*-alkanes (C8–C40, ref. 40147-U, Supelco) analysed under identical conditions. Comparisons with commercial standard compounds and published data were also used when possible. The different compounds were quantified as a relative percentage of total volatiles using relative peak area values obtained from the total ion current (TIC) values.

### Hydrosols antimicrobial activity

#### Microbial strains and growth conditions

The microbial cultures of *Staphylococcus aureus* ATCC 6538, *Escherichia coli* ATCC 8739, and *Candida albicans* ATCC 10231, stored in an ultra-freezer (ThermoFisher, STP, AS) at − 70 °C were activated in BHI broth and incubated in a bacteriological oven (Raypa, Incutterm, Barcelona, Spain) at 37 °C for 24 h. Subsequently, the inoculum was prepared in BHI broth by standardising the cell density suspension in a densitometer (DEN-1 McFarland densitometer, Grant-bio, UK) at a wavelength of 550 nm, to have a final cell density of 1.5 × 10^8^ cells/mL.

#### Antimicrobial activity assays

The susceptibility of the chosen strains to *C. leylandii*, *E. globulus*, *A. citrodora*, and *M. officinalis* hydrosols was performed using the viable cell counting method, colony forming units (CFU), as described in [66]. In brief, to prepare the samples, different concentrations of hydrosols (0.5 and 1.0 mL, representing 10 and 20% (*v/v*) of the total volume of the culture medium) were added to 4.5 mL of BHI broth with 10% of the standardised inoculum (1.5 × 10^8^ cells/mL). A control was prepared by replacing the hydrosol with sterile distilled water. The tubes were incubated at 37 °C for 24 h, followed by serial dilutions and plating on nutrient agar, for cell counting.

### Statistical analysis

The results were analysed using ANOVA statistical test with Tukey’s multiple comparison post-test using the GraphPad Prism^®^ 8.0 software (San Diego-CA, USA).

## Data Availability

Data sharing is not applicable to this article as no new data were created or analyzed in this study.
